# Reverting Antibiotic Tolerance of *Pseudomonas aeruginosa* PAO1 Persister Cells by (*Z*)-4-bromo-5-(bromomethylene)-3-methylfuran-2(5*H*)-one

**DOI:** 10.1371/journal.pone.0045778

**Published:** 2012-09-20

**Authors:** Jiachuan Pan, Ali Adem Bahar, Haseeba Syed, Dacheng Ren

**Affiliations:** 1 Department of Biomedical and Chemical Engineering, Syracuse University, Syracuse, New York, United States of America; 2 Syracuse Biomaterials Institute, Syracuse University, Syracuse, New York, United States of America; 3 Department of Civil and Environmental Engineering, Syracuse University, Syracuse, New York, United States of America; 4 Department of Biology, Syracuse University, Syracuse, New York, United States of America; The Scripps Research Institute and Sorrento Therapeutics, Inc., United States of America

## Abstract

**Background:**

Bacteria are well known to form dormant persister cells that are tolerant to most antibiotics. Such intrinsic tolerance also facilitates the development of multidrug resistance through acquired mechanisms. Thus persister cells are a promising target for developing more effective methods to control chronic infections and help prevent the development of multidrug-resistant bacteria. However, control of persister cells is still an unmet challenge.

**Methodology/Principal Findings:**

We show in this report that (*Z*)-4-bromo-5-(bromomethylene)-3-methylfuran-2(5*H*)-one (BF8) can restore the antibiotic susceptibility of *Pseudomonas aeruginosa* PAO1 persister cells at growth non-inhibitory concentrations. Persister control by BF8 was found to be effective against both planktonic and biofilm cells of *P. aeruginosa* PAO1. Interestingly, although BF8 is an inhibitor of quorum sensing (QS) in Gram-negative bacteria, the data in this study suggest that the activities of BF8 to revert antibiotic tolerance of *P. aeruginosa* PAO1 persister cells is not through QS inhibition and may involve other targets.

**Conclusion:**

BF8 can sensitize *P. aeruginosa* persister cells to antibiotics.

## Introduction

It is well documented that a small portion of a bacterial population can form metabolically inactive persister cells [1], which are not mutants with drug resistance genes, but rather phenotypic variants of the wild-type strain [2] due to unbalanced production of toxins/anti-toxins [3], [4], [5], [6] and other mechanisms related to stress response and translation inhibition [1], [7]. This subpopulation can survive the attack of antibiotics at high concentrations, and when the treatment is stopped, they can reestablish the population with a similar percentage of cells as persisters, leading to high levels of antibiotic tolerance [2]. Such intrinsic tolerance can cause chronic infections with recurring symptoms after the course of antibiotic therapy and facilitates the development and wide spread of acquired multidrug resistance through genetic mutations and horizontal gene transfer [2]. For example, high persistence mutants have been isolated from cystic fibrosis patients with lung infections [8], [9] and from patients with candidiasis [10]. Persister phenotypes have also been found in *Mycobacterium tuberculosis*, the bacterium causing chronic tuberculosis [11]. Thus, targeting persister cells may help improve infection control and prevent the development of multidrug resistant bacteria [12]. However, controlling persister cells is still an unmet challenge.

Conceivably, one approach to eliminating persister cells is to wake up this dormant population and render them to return to a metabolically active stage. These awakened cells are expected to become sensitive to antibiotics. In Gram-positive bacteria, a 17-kDa protein, named resuscitation-promoting factor (Rpf) has been discovered as a potential agent to wake up dormant cells [13]. However, a full wakeup call may cause rapid growth of a bacterial pathogen, which can lead to adverse progression of infection if the antibiotics are not admitted during the right window.

Recently, sugars such as mannitol, glucose, fructose and pyruvate have been shown to generate proton-motive force and promote the uptake of aminoglycosides by persister cells of *Escherichia coli* and *Staphylococcus aureus*, which led to enhanced susceptibility of persister cells to this class of antibiotics. The effects were observed within 1 h of incubation, less than what is required for resumption of full growth [14]. However, this approach requires relatively high concentrations of sugar (e.g. 10 mM) and is limited to aminoglycosides, but not the β-lactam antibiotic ampicillin and the fluoroquinolone ofloxacin. In addition, sugar molecules can only wake up persister cells, but cannot reduce persistence during growth (see below).

Compared to these approaches, non-metabolites that can potentiate multiple classes of antibiotics and also reduce persistence during bacterial growth may be advantageous. It is well documented that the absolute number of persister cells in a culture increases significantly when the culture enters stationary-phase and when cells form surface-attached highly hydrated structures known as biofilms [15], [16], [17]. Recent research has demonstrated that quorum sensing (QS), bacterial cell-cell signaling by sensing and responding to cell density, promotes persister formation in *Pseudomonas aeruginosa* PAO1; e.g., acyl-homoserine lactone 3-OC_12_-HSL and phenazine pyocyanin, QS signals of *P. aeruginosa*, can significantly increase the persister numbers in logarithmic phase cultures of *P. aeruginosa* PAO1 but not *E. coli* or *S. aureus* [18]. Thus, we were motivated to test if targeting such pathways may reduce persistence during bacterial growth and/or revert the antibiotic tolerance of persister cells. We found in this study that the QS inhibitor BF8 has potent activities in persister control, although our data suggest that these activities may not be through QS inhibition and BF8 may have other targets in *P. aeruginosa* (below).

## Results

### BF8 is a QS inhibitor

**Figure 1:**
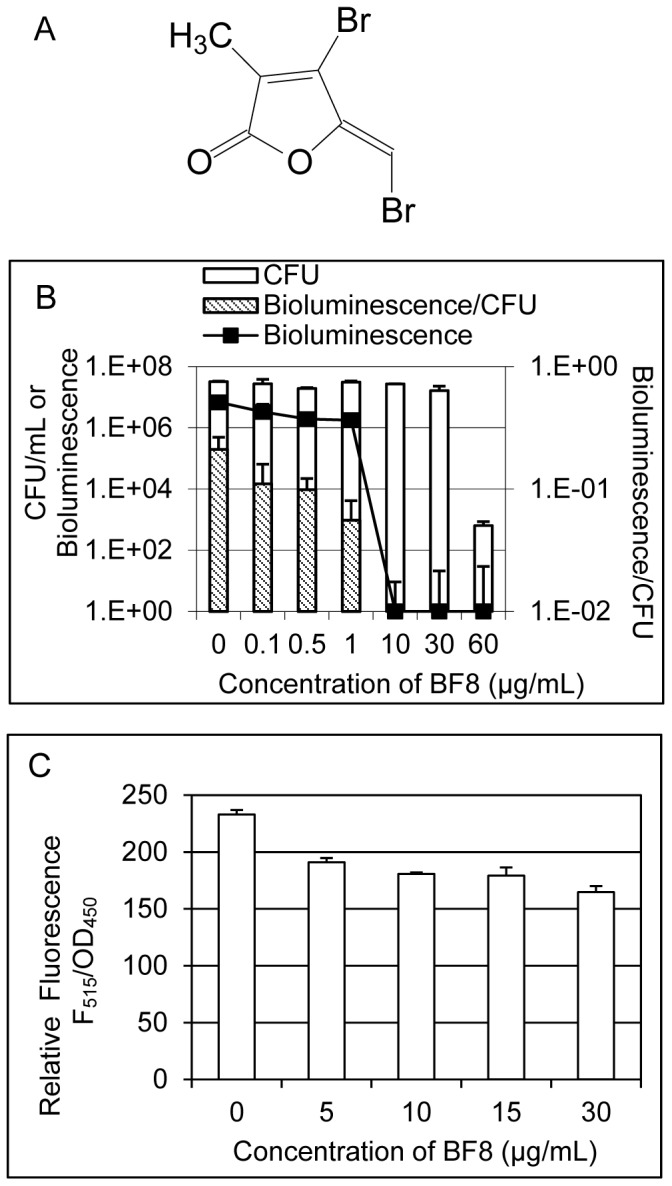
Dose-dependent inhibition of AHL-mediated QS by BF8. The structure of BF8 (A) and relative QS activities of *V. harveyi* BB886 (B) and PAO1 *lasB* reporter (C) are shown. To study the effects on QS in *V. harveyi* BB886 reporter, an overnight culture of *V. harveyi* BB886 were diluted 1∶5000 in AB medium and supplemented with different concentrations of BF8 after 5.5 h of incubation. The QS activity of each sample was characterized by normalizing the bioluminescence of the reporter *V. harveyi* BB886 with its colony forming unit (CFU) after another 1.5 h of incubation. Figure 1B shows that QS was inhibited by BF8 in a dose dependent manner. To study the effects on QS in PAO1, the reporter strain PAO1 mini-Tn5-based P*lasB*-*gfp*(ASV) was cultured till an OD_600_ of 0.8 was reached and then BF8 was added at different concentrations. The green flouresence was measured when the cultures reached stationary phase (OD_600_ around 2.7). The results show that QS in PAO1 was inhibited by BF8.

A wide variety of molecules have been discovered as quorum sensing inhibitors [19]. We reported recently that several new synthetic brominated furanones (derivatives of natural brominated furanones) are inhibitors of biofilm formation [20] and quorum sensing [21] in Gram-negative bacteria. Among these compounds, (*Z*)-4-bromo-5-(bromomethylene)-3-methylfuran-2(5*H*)-one (BF8, Figure 1A) is the most effective biofilm inhibitor of *E. coli* and *P. aeruginosa* at growth non-inhibitory concentrations [20], [21]. It is also a potent inhibitor of quorum sensing based on AI-2 [21]. In this study, the effects of BF8 on AI-1 mediated QS were studied using the reporter strain *Vibrio harveyi* BB886 (ATCC# BAA-1118) [22]. By monitoring the bioluminescence and colony forming units (CFU) of the reporter strain, BF8 was found to inhibit QS at concentrations not inhibitory to the viability of the reporter strain. For example, 10 µg/mL BF8 completely inhibited AI-1-mediated QS with no effects on the viability of *V. harveyi* BB886 (Figure 1B). To specifically test if BF8 is also an inhibitor of QS in *P. aeruginosa*, the expression of the QS-controlled toxin gene, *lasB*, in the presence of different concentrations of BF8, was characterized using the reporter *P. aeruginosa* PAO1 mini-Tn5-based P*lasB*-*gfp*(ASV) by following the procedure described previously [23]. As shown in Figure 1C, expression of *lasB* in stationary phase cultures (all around OD_600_ of 2.7) was significantly inhibited by BF8, confirming that BF8 is also an inhibitor of QS in *P. aeruginosa*.

### BF8 reduced persistence of PAO1

**Figure 2:**
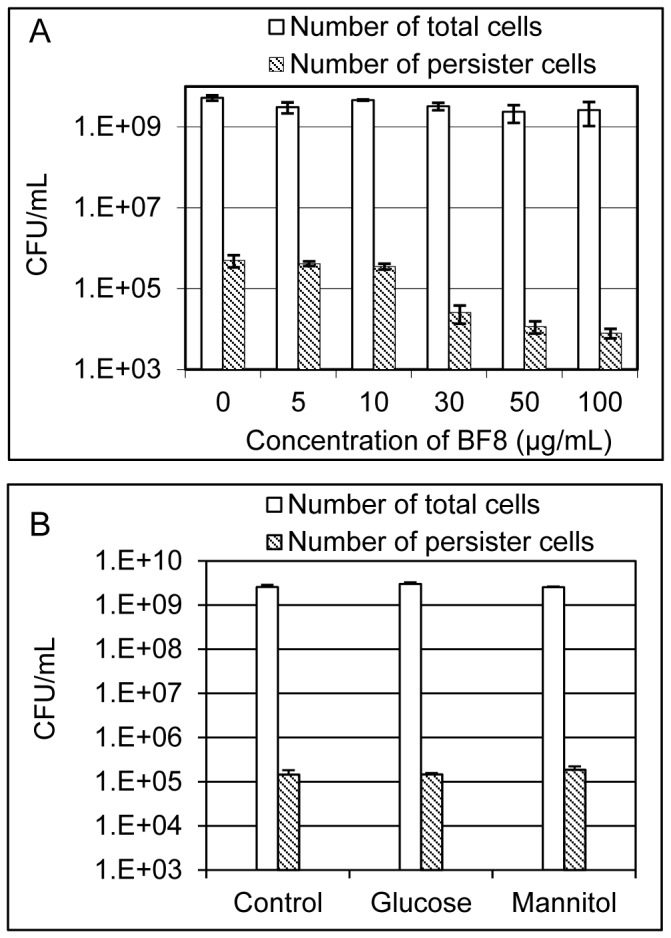
BF8 reduced persistence of PAO1 at growth non-inhibitory concentrations. (A) BF8 reduced persistence of PAO1 cultures during growth. PAO1 was cultured for 5 h in LB medium supplemented with different concentrations of BF8. The total number of viable cells and the number of persister cells after the 5 h incubation were quantified. (B) Sugars did not exhibit the same activities as BF8. The same experimental procedure was followed except that 10 mM glucose or mannitol was added instead of BF8.

To test if BF8 can control persister cells of *P. aeruginosa* PAO1 (henceforth PAO1), we studied the effects of BF8 (up to 100 µg/mL) on the viability and persistence of PAO1 during 5 h of growth in Luria Bertani (LB) medium [24]. As shown in Figure 2A, the total number of viable cells at the end of incubation was around 3.5×10^9^/mL for all the samples (one-way ANOVA, *p* = 0.122). Thus, BF8 did not affect the viability of PAO1 directly. Consistently, the MIC (minimum concentration that prevent growth overnight) of BF8 against PAO1 in LB medium was found to be higher than 200 µg/mL (Figure S1A). Interestingly, at the growth non-inhibitory concentrations, the persistence of PAO1 was significantly reduced by BF8 in a dose-dependent manner; e.g., BF8 at 100 µg/mL reduced the number of persister cells by 63 times (98.4% reduction) compared to the untreated control (one-way ANOVA, *p* = 0.0006). The reduction of persistence could lead to better efficacy of antibiotics [e.g., ciprofloxacin (Cip) as shown in Figure 2A] and help prevent the development of antibiotic resistance. To our best knowledge, this is the first compound known to reduce bacterial persistence during normal growth.

Sugars have been reported to sensitize persisters to antibiotics [14] and Wang et al. [25] reported that relatively high concentrations of fructose and glucose reduced the expression of QS-related gene *pqs*A and the production of extracellular proteases and pyocyanin in *P. aeruginosa*. To test if sugars can also reduce persistence of PAO1 under our experimental condition, we repeated the above experiment using 10 mM D-glucose and D-mannitol instead of BF8. It was found that, unlike BF8, incubation with neither of these sugars affected persistence (Figure 2B, one-way ANOVA, *p* = 0.43). These data suggest that persister control by BF8 is through a different mechanism than that by sugars.

### BF8 reverted the antibiotic tolerance of PAO1 persister cells

**Figure 3:**
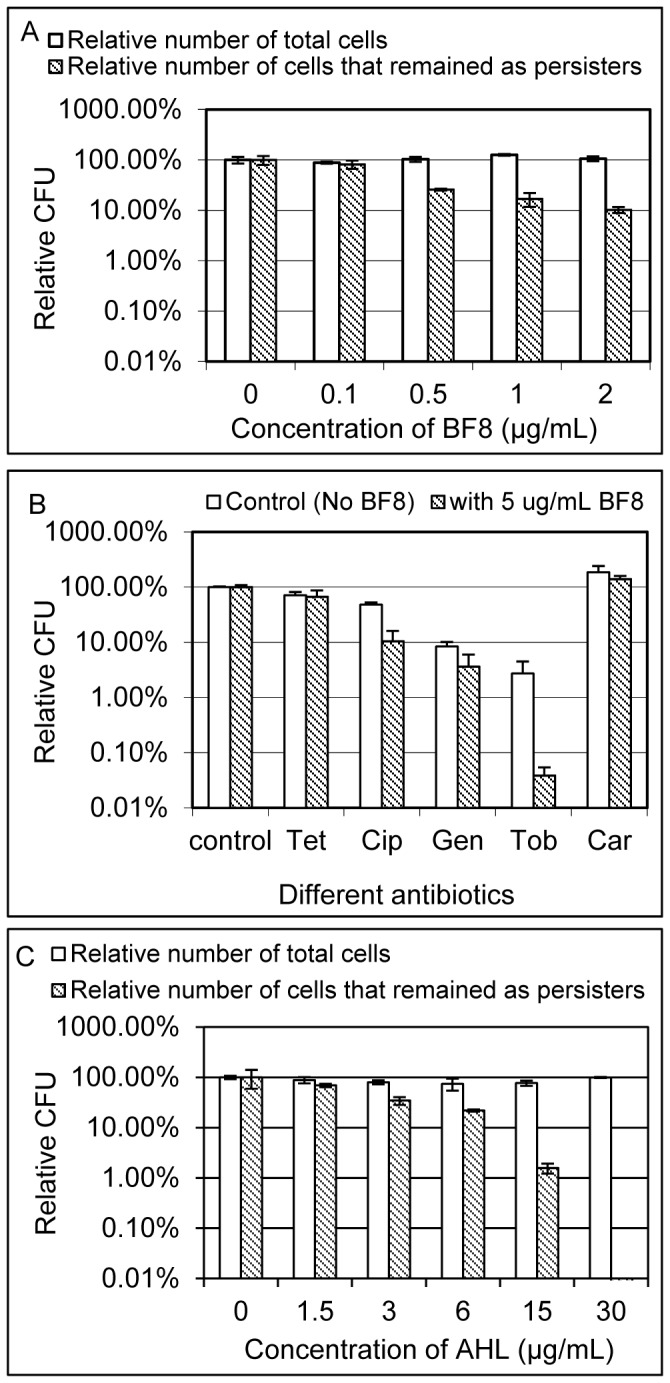
BF8 and 3-oxo-C_12_-HSL reverted antibiotic tolerance of isolated PAO1 persister cells. The total number of viable cells and the number of cells that remained as persisters of untreated controls in each graph were normalized as 100% for the convenience of data comparison across the three experiments. (A) BF8 reverted Cip tolerance of isolated PAO1 persister cells. The harvested PAO1 persister cells were treated with different concentrations of BF8 for 2 h in 0.85% NaCl solution and the viability of PAO1 was evaluated by counting CFU. A portion of each sample was then treated with 200 µg/mL Cip to count the number of PAO1 cells that remained as persisters. The start number of persisters was 2.1×10^6^±3.1×10^5^/mL. (B) Antibiotic susceptibility of PAO1 persister cells treated with and without 5 µg/mL BF8. Persisters were isolated and treated with or without 5 µg/mL BF8 in 0.85% NaCl solution for 2 h. The treated cells were then incubated with different antibiotics for 3.5 h to test antibiotic susceptibility. PAO1 persisters were found to be sensitized to Tob and Cip. The start number of persisters was 2.3×10^6^±5.7×10^4^/mL. (C) The QS signal 3-oxo-C_12_-HSL also sensitized PAO1 persisters to Cip. The same procedure as shown in Figure 3A was followed except that 3-oxo-C_12_-HSL was tested instead of BF8. The start number of persisters was 2.0×10^6^±4.0×10^5^/mL.

In addition to reducing persistence during PAO1 growth, BF8 was also found to revert the antibiotic tolerance of isolated persisters. As shown in Figure 3A, treatment with BF8 at all tested concentrations (0.1, 0.5, 1, and 2 µg/mL) increased the susceptibility of persister cells to Cip. For example, although BF8 at 0.5 µg/mL did not affect the viability of persister cells, the antibiotic tolerance of persister cells was reverted since 74.1 ±1.1% of persister cells became sensitive to Cip compared to the untreated control (One-way ANOVA, *p* = 0.0005). The effects on persistence reduction increased to 89.8±1.4% when BF8 was added at 2 µg/mL (one-way ANOVA, *p* = 0.0013) (Figure 3A). At higher concentrations, however, BF8 was found to be cidal to PAO1 persister cells. For example, treatment with 10 µg/mL BF8 led to significant killing of PAO1 persister cells (data not shown), suggesting that a threshold concentration may exist between growth non-inhibitory reversion of persistence and cidal effects on persister cells. Consistently, BF8 at 2 and 5 µg/mL did not affect the viability of regular PAO1 cells in stationary phase (one-way ANOVA, *p* = 0.7975 and p = 0.8572, respectively, Figure S1B). It appeared to be cidal to regular cells at 10 µg/mL or higher concentrations (Figure S1B); while the MBC (the minimum concentration that reduces viability by 99.9% [26], [27]) was found to be higher than 30 µg/mL (the highest concentration tested). Overall, the above finding shows that BF8 can revert persistence at concentrations that do not affect the viability of both persister and regular cells of PAO1 (up to 5 µg/mL under our experimental condition).

We chose 0.85% NaCl solution rather than LB medium to test the effects on isolated persisters because NaCl solution itself does not contain carbon source, allowing the effects on viability to be tested specifically. The concentrations of BF8 that exhibited activities were significantly lower in 0.85% NaCl solution than those in LB medium (to test persistence during growth as described above), presumably because LB medium contains proteins and other large molecules that may bind to BF8 and decrease its effective concentration. It is also worth noticing that the persister numbers are higher in Figures 3 (start CFU/mL as 2.1×10^6^±3.1×10^5^ in 3A, 2.3×10^6^±5.7×10^4^ in 3B, and 2.0×10^6^±4.0×10^5^ in 3C) than those in Figures 2 (5.0×10^5^±1.7×10^5^/mL for the control) because the persister cells in Figure 3 were isolated from overnight cultures (known to have higher persistence [28], [29]) and those in Figure 2 were isolated from growing cultures.

It is interesting that, unlike sugars which can only potentiate aminoglycosides [14], BF8 was found to restore susceptibility of PAO1 persister cells to both ciprofloxacin and tobramycin (from two different classes of antibiotics). In total, five antibiotics were tested to evaluate the effects on antibiotics with different targets including protein synthesis [tetracycline (Tet), gentamicin (Gen) and tobramycin (Tob)], cell wall synthesis [carbenicillin (Cab)], and functions of DNA gyrase (Cip). In addition to Cip (*t* test, *p* = 0.0095), BF8 at 5 µg/mL was also found to potentiate Tob (*t* test, *p* = 0.0271), while the effects on Tet (*t* test, *p* = 0.4096), Gen (*t* test, *p* = 0.0771), and Car (*t* test, *p* = 0.1976) were not statistically significant (Figure 3B).

Since QS is known to stimulate persister formation in PAO1 [18] and BF8 is a QS inhibitor, we further tested if persister controlled by BF8 can be relieved by the QS signal. It was interesting to find that addition of 3-oxo-C_12_-HSL (Sigma-Aldrich, St. Louis, MO, USA) was not able to reduce the inhibitory effects of BF8 (Figure 3C). Instead, 3-oxo-C_12_-HSL was also found to sensitize isolated persisters to Cip in a dose dependent manner. For example, after treatment with 30 µg/mL 3-oxo-C_12_-HSL for 2 h, nearly all the isolated persisters were killed by 200 µg/mL Cip (Figure 3C). Interestingly, this AHL was found previously to promote PAO1 persister formation in exponential phase (different experimental condition than described here) [18]. Thus, this QS signal may have different effects on PAO1 persisters under different conditions. These findings suggest that, although BF8 is a QS inhibitor, the activities of BF8 to sensitize PAO1 persisters to antibiotics is not through QS inhibition and there are other targets of BF8 in PAO1 persister cells.

### Effects of BF8 on PAO1 biofilms and associated persister cells

**Figure 4:**
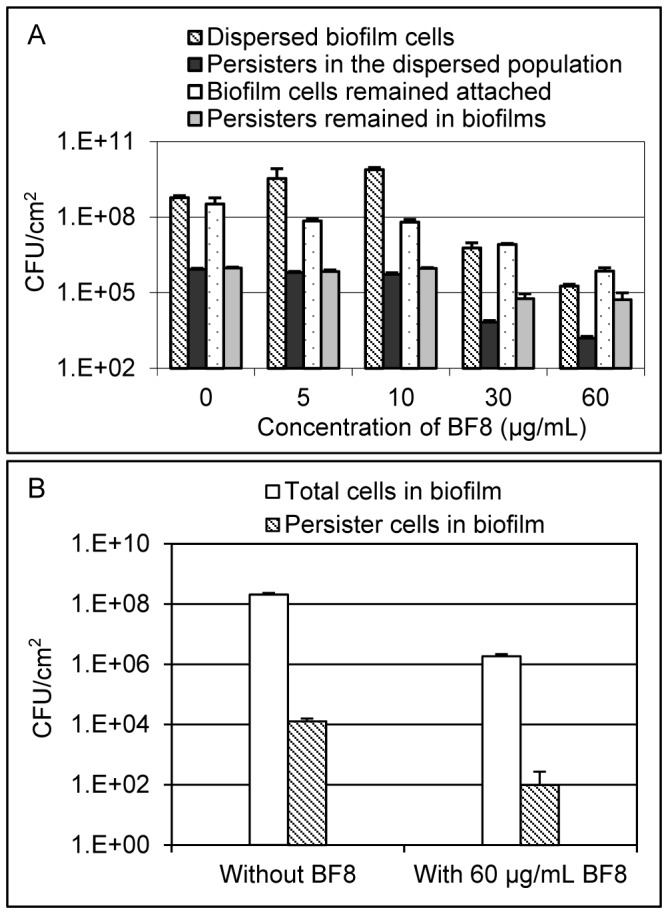
BF8 is effective against PAO1 biofilms. (A) BF8 dispersed biofilm and reduced persistence in both biofilm and the detached population. The biofilms were treated with BF8 at different concentrations for 24 h in 0.85% NaCl solution. (B) BF8 inhibited biofilm formation and reduced the number of persister cells in biofilms. BF8 was added at inoculation and the biofilms were cultured for 18 h.

Compared to planktonic cells, surface-attached bacterial biofilms are more challenging to microbial control since they are up to 1000 times more tolerant to antibiotics than planktonic cells and are known to harbor a high percentage of persister cells [1], [30]. To understand if BF8 can also control persisters in biofilms, we treated 18-h PAO1 biofilms formed on 304L stainless steel coupons with different concentrations of BF8 for 24 h. Both the planktonic (detached cells) and biofilm populations that remained attached were analyzed to evaluate the viability and persistence of PAO1 with and without BF8 treatment. As shown in Figure 4A, BF8 dispersed established biofilms and reduced the number of persister cells in both biofilm and detached population. For example, the number of viable cells remained attached after treatment was reduced by 5 µg/mL BF8 from 3.3×10^8^±1.7×10^8^/cm^2^ to 7.1×10^7^±1.4×10^7^/cm^2^ (one-way ANOVA, *p* = 0.0025). Among the cells that remained attached, the number of persisters was reduced from 9.6×10^5^±9.1×10^4^/cm^2^ to 7.0×10^5^±1.1×10^5^/cm^2^ (one-way ANOVA, *p* = 0.002). At concentrations up to 10 µg/mL, BF8 did not exhibit cidal effects but reduced the percentage of persister cells (0.14±0.01% without BF8 vs. 0.013±0.002% with 10 µg/mL BF8, one-way ANOVA, *p* = 0.0002) in the detached population (the total number of cells in suspension increased compared to the control due to detachment); while at high concentrations, BF8 appeared to be cidal to both regular and persister cells. For example, treatment with 60 µg/mL BF8 for 24 h led to 94.2±5.1% reduction of viable persister cells remained on the surface (one-way ANOVA, *p* = 0.0004), although the persisters/regular cells ratio in biofilms was not reduced by BF8 (Figure 4A). In addition to the effects on established biofilms, BF8 at 60 µg/mL added at inoculation was also found to inhibit PAO1 biofilm formation (incubated for 18 h) by 99.1×0.2% (*t* test, *p* = 0.0001) and reduced the number of biofilm-associated persisters by 99.2±1.3% (*t* test, *p* = 0.001) (Figure 4B).

### DNA microarray analysis

It was an interesting finding that BF8 can render persisters sensitive to the antibiotics targeting 30S ribosome RNA (Tob), and topoisomerase (Cip). The capability to sensitize persister cells to antibiotics that target both DNA replication and protein synthesis suggests that BF8 may have made the cells leave the persister stage. To obtain a deeper insight at the genetic level, we investigated the effects of BF8 on sensitization of PAO1 persister cells using DNA microarrays. The gene expression profiles of PAO1 persister cells treated with and without BF8 at 1 µg/mL for 1 h were compared in triplicate. We chose this effective, but relatively low, concentration of BF8 (as shown in Figure 3A) so that the most important genes induced by BF8 can be identified. The persister cells were isolated by killing regular cells with 200 µg/mL Cip for 3.5 h. Because average half-life of bacterial mRNA is only a few minutes [31], we expect that the mRNA in dead cells should be degraded when the cells were harvested. Consistently, we found that 85.5% of the mRNA of the house-keeping gene *proC* was degraded in the persister sample compared to the sample before Cip treatment (Figure S3). Furthermore, since the identical persister cell samples were used for both the control (no BF8) and test (with BF8), only the differentially expressed genes in live cells are expected to be seen in the microarray data.

In total, 28 genes were consistently induced by BF8 by more than 2 fold compared to the control in all three biological replicates (see Table S1 for the full list). In comparison, although a relatively small set of repressed genes was seen in each set, no gene was significantly repressed in all three sets mostly due to low expression ratios in some dataset(s) (test/control <2.0). This is possibly because persister cells only have low level expression of essential genes due to their dormant nature [32], [33]. To validate the DNA microarray results, we conducted RNA slot blotting for five representative genes including one unchanged gene (PA4943) and 4 induced genes (PA3523, PA2931, PA0182 and PA4167). The results of all blots were consistent with the microarray data (Table S2). The consistently induced genes encode oxidoreductases (PA4167, PA1334, PA0182, PA2932, PA2535, PA3223, PA1127), transcriptional factors (PA4878, PA1285, PA3133, PA2196), and hypothetical proteins (PA4173, PA0741, PA1210, PA3240, PA2575, PA0565, PA2580, PA2610, PA2839, PA0422, PA1374, PA2691). Since many reductases are involved in metabolism, our DNA microarray data indicate that some cellular activities or membrane functions of PAO1 persisters can be induced by low concentrations of BF8. In addition, the gene PA2931 was induced by 11 times. This gene encodes a repressor of Cif, a *P. aeruginosa* toxin that causes degradation of the cystic fibrosis transmembrane conductance regulator (CFTR) in mammalian cells [34], [35]. The induction of PA2931 indicates that BF8 can potentially repress the pathogenicity of PAO1. No QS genes were found to be differentially expressed by BF8. This is not surprising since persister cells are relatively dormant and are not expected to have QS activities. This finding further supports that persister control by BF8 involves other pathways and confirms that the mRNAs of differentially expressed genes were indeed from persister cells.

## Discussion

In this study, we show that BF8 can act synergistically with antibiotics to enhance killing of *P. aeruginosa* PAO1 persister cells. Although more work is needed to reveal the exact mechanism, the restoration of antibiotic susceptibility of PAO1 persister cells at growth non-inhibitory concentrations by BF8 is nevertheless interesting. The DNA microarray data suggest that some reductases and proteins for small molecule transfer were induced by BF8. We hypothesize that interaction between BF8 (at growth non-inhibitory concentration) and cell membrane proteins can interrupt specific cellular functions, which led to increase in activities of transport proteins and reductases. Such response should require energy and thus may influence the physiological stage of persister cells and restore their susceptibility to antibiotics. Such effects may be mechanistically different from natural wakeup when the persister cells are supplied with a new medium. Further study on bacterial membrane potential and metabolism with and without BF8 (at growth non-inhibitory concentrations) can help test this hypothesis. In an earlier work, Shah et al. [33] compared gene expression in regular cells and persisters of *E. coli* and found that around 5% of genes are differentially expressed between these two populations. A number of genes involved in toxin-antitoxin module proteins rather than stationary-phase-specific functions were induced in persisters compared to regular cells. In our PAO1 microarray data, however, only a short list of genes was induced by BF8, which is different from that of regular cells vs. persister cells [33]. These data confirmed that treatment with BF8 was not leading to a full wakeup. Because the cells only activate certain functions, such treatment may act as a partial wakeup and can be advantageous compared to a full wakeup that leads to normal cell growth and potentially higher virulence. Molecules with such activities may have a good opportunity to be applied either before or together with antibiotics to clean infections, without a specific window required for antibiotics to be administered.

To be applied for disease control, it is important to evaluate the safety and efficacy of BF8 *in vivo*. This is part of our ongoing work. Nevertheless, some other brominated furanones have been shown to be safe and effective in animal models such as shrimps [36] and mice [37]. For example, furanone C-30 has been shown to reduce the virulence of *P. aeruginosa* and help clear infection from the lungs of mice [37]. The activities of persister control found in the present study bring new opportunities to develop more effective therapies based on this class of compounds.

In summary, the results described above indicate that BF8 can reduce persistence during the growth of PAO1 and can also restore the susceptibility of isolated persister cells to antibiotics. This appears to be a promising advantage of BF8 for persister control. The exact targets of BF8 and the chemical nature of such interaction are unknown and are a goal of our ongoing work. It is important to understand if there are a set of specific membrane proteins, activation of which can lead to higher antibiotic susceptibility; and if a subset of such proteins is sufficient for the observed activities. Better understanding of the underlying mechanism will help develop more effective methods to control bacterial persistence and associated chronic infections.

## Materials and Methods

### Furanone synthesis

(*Z*)-4-bromo-5-(bromomethylene)-3-methylfuran-2(5*H*)-one (BF8) was synthesized as described previously [20], dissolved in absolute ethanol as 60 mg/mL, and stored at 4°C until use. Briefly, Br_2_ (6.22g, 38.9 mmol) in dichloromethane (20 mL) was added dropwise into a flask containing 2.53 g (19.5 nmol) alpha-methyllevulinica acid in 20 mL dichloromethane. The mixture was stirred at 35∼40°C till all the alpha-methyllevulinica acid reacted (based on TLC test); and then the reaction was interrupted by adding ice (∼200 mL). The mixture was extracted with dichloromethane three times (80 mL each), washed with Na_2_S_2_O_3_ (1 M, 100 mL) to remove residue Br_2_, dried with anhydrous sodium sulfate (30 min), filtered with cotton, and then purified by removing solvent using a rotary evaporator. The crude bromo keto acid was added with concentrated H_2_SO_4_ (98%, 10 mL) and the mixture was heated in an oil bath at 110°C till all the crude keto reacted (by checking on TLC plates). The raw product was poured into a beaker with 200 mL ice to stop the reaction. The mixture was extracted with dichloromethane three times (50 mL each), washed once with 80 mL H_2_O and dried using a rotary evaporator. BF8 was further purified from other impurities using column chromatography (dichloromethane: hexanes  = 1: 4). The structure of BF8 was confirmed using ^1^H-NMR by comparing with reported data [20].

### Bacterial strain and growth media

Planktonic PAO1 cultures were routinely grown in Luria-Bertani (LB) medium [24] which contains 10 g/L NaCl, 10 g/L tryptone and 5 g/L yeast extract. To minimize the variation in the level of persistence, all overnight cultures of PAO1 were inoculated using single-use glycerol stocks (disposed after use to avoid freeze and thaw) prepared from the same batch of PAO1 overnight culture. The *P. aeruginosa* QS reporter strain PAO1 mini-Tn5-based P*las*B-*gfp*(ASV) [23] was routinely grown in modified LB medium [23] containing 10 g/L trypton, 5 g/L yeast extract, and 4 g/L NaCl. Overnight cultures of *V. harveyi* BB886 were grown in LM medium [38] containing 10 g/L tryptone, 5 g/L yeast extract, and 20 g/L NaCl. PAO1 biofilms were cultured in M63 medium [39] containing 13.6 g/L KH_2_PO_4_, 2 g/L (NH_4_)_2_SO_4_, and 0.5 mg/L FeSO_4_·7H_2_O, pH 7, supplemented with 0.3% glucose, 1 mM MgSO_4_ and 0.5% casamino acids.

### Persister isolation

Treatment with Cip up to 50 µg/mL for 3.5 h has been used to isolated PAO1 persister cells previously [18]. We confirmed recently that treatment with 50 µg/mL Cip for 3.5 h is also sufficient to kill regular cells of our PAO1 strain since no additional killing was observed with Cip concentration up to 200 µg/mL (the highest concentration tested [40]. To further confirm that the treatment time is sufficient, we also tested the killing with 200 µg/mL Cip during 6.5 h of incubation. As shown in Figure S2, no additional killing was observed with incubation beyond 1.5 h. Given these results, we chose incubation for 3.5 h with 200 µg/mL Cip to ensure the complete elimination of regular cells. After Cip treatment (200 µg/mL, 3.5 h) of 18-h PAO1 overnight cultures, the surviving persister cells were washed twice with 0.85% NaCl solution to remove residual antibiotics, and then resuspended in 0.85% NaCl solution. The isolated persister cells were then used for different treatments as described below. The cells after each treatment were further treated by supplementing with 200 µg/mL Cip and incubating for 3.5 h. Then the samples were washed three times with 0.85% NaCl solution to quantify the number of cells that remained as persisters. The drop plate method described by Chen et al. [41] was followed to count colony forming units (CFUs).

### Effect of BF8 on AHL-mediated QS in the reporter strain *V. harveyi* BB886

A *V. harveyi* BB886 overnight culture was used to inoculate subcultures in AB medium [22]. BF8 was added at different concentrations (0, 0.1, 0.5, 1, 10, 30, 60 µg/mL) after 5.5 h of growth at 37°C with 200 rpm shaking. The incubation continued for another 1.5 h. Then the bioluminescence was measured using a luminometer (20/20n, Turner Design, Sunnyvale, CA, USA). Meanwhile, the CFU of reporter cells was determined using drop method with LM agar plates [22], [38] after washing the cells with 2% NaCl solution. This experiment was performed with two biological replicates and 6 replicates on drop plates were counted for each CFU data point.

### Effect of BF8 on QS in PAO1

A overnight culture of the QS reporter strain PAO1 mini-Tn5-based P*las*B-*gfp*(ASV) [23] was used to inoculate subcultures in modified LB medium [23]. When the subcultures reached OD_600_ of 0.8, BF8 was added at different concentrations (0, 5, 10, 15, and 30 µg/mL). Green fluorescence and OD_450_ was measured when OD_600_ reached around 2.7 by following the previously described protocol [23] to evaluate the effects on QS in PAO1. This experiment was conducted in duplicate.

### Effects of BF8 on persistence of PAO1

A PAO1 overnight culture was used to inoculate subcultures (each contained 5 mL LB medium) to an OD_600_ of 0.05, which were then supplemented with different concentrations of BF8 (0, 5, 10, 30, 50 and 100 µg/mL). The amount of ethanol (solvent of BF8 stock solutions) was adjusted to be the same for each sample to eliminate any solvent effect. Samples were taken after 5 h of incubation at 37°C with shaking at 200 rpm to count CFU. Meanwhile, the remaining portion of each sample was added with 200 µg/mL Cip and incubated for 3.5 h at 37°C. The samples were then analyzed to quantify the number of persister cells by counting CFU. This experiment was performed with two biological replicates and 6 replicates on drop plates were counted for each CFU data point.

### Effects of D-glucose and D-mannitol

*P. aeruginosa* PAO1 subcultures were inoculated with an overnight culture to an initial OD_600_ of 0.05 in LB medium. The subcultures were supplemented with 10 mM D-glucose, 10 mM D-mannitol or without sugar (control). The total number of viable cells and the number of persister cells were quantified as described in the experiment of BF8 above. This experiment was conducted with two biological replicates and 5 replicates on drop plates were counted for each CFU data point.

### Effects of BF8 on antibiotic susceptibility of isolated persister cells

Persisters were isolated from overnight cultures as described above. After dilution by 50 times with 0.85% NaCl solution, the persisters were challenged with different concentrations of BF8. Ethanol (the solvent used for making BF8 stock solutions) was adjusted to be the same in all samples to eliminate any solvent effect. After incubation for 2 h at 37°C with shaking at 200 rpm, 1 mL of each sample was taken and washed three times with 0.85% NaCl to quantify the total number of viable cells by counting CFU. The remaining portion of each sample was further tested to quantify the number of cells that remained as persisters as described above. This experiment was conducted with two biological replicates and 5 replicates on drop plates were counted for each CFU data point.

### Synergy with other antibiotics

Persisters were isolated from overnight cultures as described above, and then incubated in 0.85% NaCl for 2 h at 37°C with shaking at 200 rpm in the absence or presence of 5 µg/mL BF8. The amount of ethanol was adjusted to be the same in all samples to eliminate any solvent effect. After incubation, 1 mL of BF8 treated persister samples and BF8-free controls were added with and without different antibiotics [25 µg/mL tetracycline (Tet), 25 µg/mL gentamicin (Gen), 25 µg/mL tobramycin (Tob), 500 µg/mL carbenicillin (Car), 25 µg/mL ciprofloxacin (Cip)] and incubated for another 3.5 h at 37°C with shaking at 200 rpm. The antibiotic treated persisters were then washed three times with 0.85% NaCl solution to remove antibiotics and plated on LB plates to evaluate the killing by antibiotics by counting CFU. This experiment was conducted with two biological replicates and 5 replicates on drop plates were counted for each CFU data point.

### Effects of *N*-(3-Oxododecanoyl)-*L*-homoserine lactone (3-oxo-C_12_-HSL)

This experiment was conducted by following the same protocol as that for the effects of BF8 on isolated persister cells described above. The QS signal 3-oxo-C_12_-HSL was tested at 0, 1.5, 3, 6, 15, and 30 µg/mL. This experiment was conducted with three biological replicates and 5 replicates on drop plates were counted for each CFU data point.

### Effects of BF8 on persister cells in established biofilms

*P. aeruginosa* PAO1 overnight cultures in LB medium were used to inoculate subcultures in M63 medium to an OD_600_ of 0.05 in glass petri dishes containing 2 cm ×1 cm 304L stainless steel coupons. After 18 h of incubation, the coupons with established biofilms were transferred to a 12 well plate (Becton Dickinson, Franklin Lakes, NJ, USA). Each well contained 4 mL of 0.85% NaCl solution supplemented with different concentrations of BF8 (0, 5, 10, 30, 60 µg/mL). The biofilm samples in 12 well plates were incubated at 37°C for 24 h without shaking. One mL of medium with detached cells was then sampled from each well, washed three times with 0.85% NaCl solution and plated on LB agar plates to determine the viability of PAO1 cells by counting CFU. Meanwhile, 1 mL of medium with detached cells was sampled, added with 200 µg/mL Cip, and incubated for 3.5 h at 37°C to isolate persister cells. Then the samples were washed three times with 0.85% NaCl solution and plated on LB agar plates to determine the number of persister cells by counting CFU. To collect the biofilm cells, the coupons were transferred to 15 mL falcon tubes, each containing 5 mL 0.85% NaCl solution. The biofilm cells were collected by vortexing the coupons for 1 min and sonicating (Ultrasonic cleaner Model No B200, Sinosonic Industrial Co., Ltd, Taipei Hsien, Taiwan) for 1 min (repeat once) [42]. Collected biofilm cells were plated on LB plates to count CFU and the rest of the samples were treated with 200 μg/mL Cip for 3.5 h at 37°C for persister isolation. The isolated biofilm-associated persister cells were washed three times and plated on LB agar plates to count CFU. This experiment was conducted with three biological replicates and 5 replicates on drop plates were counted for each CFU data point.

### Effects of BF8 on PAO1 biofilm formation

Biofilms were formed on 2 cm ×1 cm 304L stainless steel coupons in M63 medium. The biofilm cultures with and without 60 µg/mL BF8 (but with the same amount of the solvent ethanol) were inoculated with an overnight culture to an initial OD_600_ of 0.05. After 18 h of incubation at 37°C without shaking, the coupons were gently washed with 0.85% NaCl solution three times to remove unattached planktonic cells. The total number of biofilm cells and the number of persisters were quantified as described above. This experiment was conducted with three biological replicates and 5 replicates were counted for each CFU sample using drop plate method.

### DNA microarray analysis

Persister cells were harvested from 18-h cultures of PAO1 (100 mL each) using the same methods as described above. The isolated persister cells were resuspended in 0.85% NaCl solution supplemented with 1 µg/mL (3.7 µM) BF8 or with the same amount of ethanol (4.17 µL, to eliminate the solvent effects). After incubation at 37°C for 1 h, treated persister cells were collected by centrifugation at 10,000 rpm for 5 min at 4°C, transferred to 2 mL pre-cooled microcentrifuge tubes and frozen instantly in an ethanol-dry ice bath. The cell pellets were stored at −80°C until RNA isolation.

To isolate the total RNA, the harvested PAO1 cells were lysed by beating at 4,800 oscillations/min using a mini-bead beater (Biospec Products Inc., Bartlesville, OK, USA) after adding 0.5 mm glass beads, 900 µL RLT buffer and 1% 2-Mercaptoethanol. The total RNA was extracted using RNeasy Mini Kit (Qiagen, Austin, TX, USA) with on-column DNase treatment (RNase-Free DNase Set, Qiagen). The RNA samples were sent to the DNA microarray Facilities at SUNY Upstate Medical University for microarray (*P. aeruginosa* Genome Array, Affymetrix, Santa Clara, CA, USA) hybridization. A total of three biological replicates were tested. Using the GeneChip Operating Software (MAS 5.0), genes with a *p*-value of less than 0.0025 or greater than 0.9975 were considered statistically significant based on Wilcoxon signed rank test and Tukey Byweight. To ensure the significance of microarray data, an additional criterion was applied to only select the genes with an expression ratio of 2 or higher from this group as induced and repressed genes. Microarray data has been deposited in Gene Expression Omnibus (GEO: GSE36753), compliant with Minimum Information About a Microarray Experiment (MIAME) guidelines.

### RNA slot blotting

A total of five genes were tested including PA3523, PA2931, PA0182, PA4167 and PA4943. Primers were designed to include only small inner regions, varying from 368 bp to 448 bp, of these genes. Hybridization probes were labeled with DIG-dUTP (PCR DIG Probe Synthesis Kit, Roche, Mannheim, Germany) in PCR reactions by following the manufacturer's protocol. Total RNA was isolated as described in the DNA microarray section above. The blotting and signal detection were conducted as we described previously [43].

### Q-PCR analysis

To verify if killing of PAO1 cells by Cip led to mRNA degradation in the dead cells, the expression levels of the house-keeping gene *proC* were quantified using Q-PCR. Total RNA was extracted from overnight PAO1 cells before and after 3.5 h of treatment with 200 µg/mL Cip. Then, 200 ng total RNA was taken from each sample to perform cDNA synthesis by using iScript cDNA synthesis kit (Bio-Rad, Hercules, CA, USA). Two primers were used in Q-PCR including the forward primer CGTGGTCGAGTCCAACGCCG and the reverse primer GCGTCGGTCATGGCCTGCAT. Relative expression ratios were calculated from triplicate reactions.

### Minimal inhibitory concentration (MIC) of BF8

Subcultures of PAO1 were inoculated from an 18-h overnight culture to an OD_600_ of 0.05. BF8 was added at different concentrations (0–200 µg/mL) and OD_600_ at this time point was measured. After 24 h incubation at 37°C, the presence and absence of growth were checked by comparing the OD_600_ before and after incubation. The experiment was performed with six biological replicates.

### Minimal bactericidal concentration (MBC) of BF8

An 18-h overnight culture of PAO1 was washed and diluted with 0.85% NaCl solution to an OD_600_ of 0.05 supplemented with different concentrations of BF8 (0–30 µg/mL). After 2 h of incubation at 37°C in culture tubes, the treated cells were washed and diluted with 0.85% NaCl solution to count CFU using drop plate method. The experiment was performed with 2 biological replicates.

### Effects of Cip treatment time on PAO1 killing

An overnight culture of PAO1 was incubated with 200 µg/mL Cip at 37°C with 200 rpm shaking. At different incubation time point (1.5 h–6.5 h), Cip treated cells was sampled, washed by three times, diluted and plated on LB agar plates to determine CFU. The experiment was performed with 2 biological replicates.

## Supporting Information

Figure S1 **Effects of BF8 on growth and viability of** ***P. aerugionsa*** **PAO1.** (A) Effects on growth. LB medium was inoculated with overnight *P. aeruginosa* PAO1 cultures to an OD_600_ of 0.05. BF8 was added at different concentrations (0–200 µg/mL) and the presence and absence of growth were followed after 24 h of incubation at 37°C. The results indicate that none of the tested concentrations was sufficient to inhibit growth completely. Therefore the MIC was found to be higher than 200 µg/mL in LB medium. (B) Effects on viability. An 18-h overnight culture of PAO1 was washed and diluted with 0.85% NaCl solution to an OD_600_ of 0.05 supplemented with different concentrations of BF8 (0–30 µg/mL). After 2 h of incubation, the number of viable cells was determined by counting CFU. The results indicate that none of the tested concentrations was sufficient to kill more than 99.9% of PAO1 (Figure S1B). Therefore the MBC (minimum concentration that reduce viability by 99.9% [26], [27]) in 0.85% NaCl solution was found to be higher than 30 µg/mL.
(TIF)

Figure S2 **Effects of Cip treatment time on PAO1 killing.** An 18-h overnight culture of PAO1 was treated with 200 µg/mL Cip for different lengths of time to determine the required treatment time for persister isolation.
(TIF)

Figure S3 **Transcription level of the housekeeping gene,** ***proC*** **, from total cells (before Cip treatment) and persister cells quantified with Q-PCR.** The persister cells were isolated following the same procedure as described in the manuscript. The cells before and after Cip treatment were used to isolate total RNA and compare the transcription levels of *proC*. The persister cell sample was found to have 85.5% less *proC* compared to that of total cells before Cip treatment.
(TIF)

Table S1 **List of BF8-inducded genes in PAO1 persister cells.** A total of three biological replicates were tested. The genes induced by more than 2 fold in all three data sets are listed below.
(DOCX)

Table S2 **The primers used in RNA slot blotting and the blotting results.** PA4943 was unchanged based on DNA microarray data. All the other 4 genes were induced by BF8 based on microarray results.
DOCX
