# Supplementary figures and images for: Reverting Antibiotic Tolerance of Pseudomonas aeruginosa PAO1 Persister Cells by (Z)-4-bromo-5-(bromomethylene)-3-methylfuran-2(5H)-one
Source: PLoS One. 2012 Sep 20;7(9):e45778. doi: 10.1371/journal.pone.0045778 (PMC3447867; doi:10.1371/journal.pone.0045778)

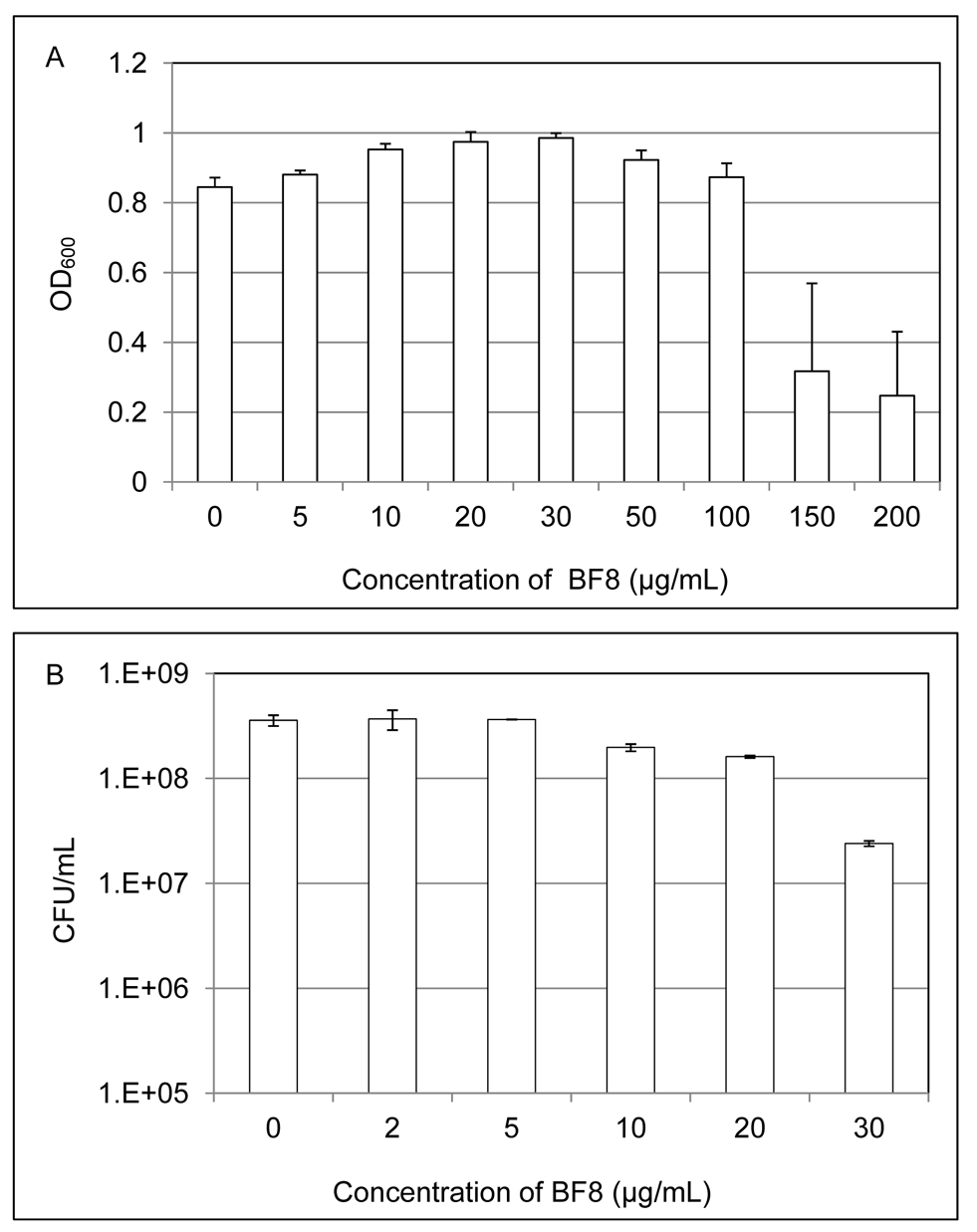

Supplement: Figure S1 — Effects of BF8 on growth and viability of P. aerugionsa PAO1. (A) Effects on growth. LB medium was inoculated with overnight P. aeruginosa PAO1 cultures to an OD600 of 0.05. BF8 was added at different concentrations (0–200 µg/mL) and the presence and absence of growth were followed after 24 h of incubation at 37°C. The results indicate that none of the tested concentrations was sufficient to inhibit growth completely. Therefore the MIC was found to be higher than 200 µg/mL in LB medium. (B) Effects on viability. An 18-h overnight culture of PAO1 was washed and diluted with 0.85% NaCl solution to an OD600 of 0.05 supplemented with different concentrations of BF8 (0–30 µg/mL). After 2 h of incubation, the number of viable cells was determined by counting CFU. The results indicate that none of the tested concentrations was sufficient to kill more than 99.9% of PAO1 (Figure S1B). Therefore the MBC (minimum concentration that reduce viability by 99.9% [26], [27]) in 0.85% NaCl solution was found to be higher than 30 µg/mL. (TIF) [file pone.0045778.s001.tif]

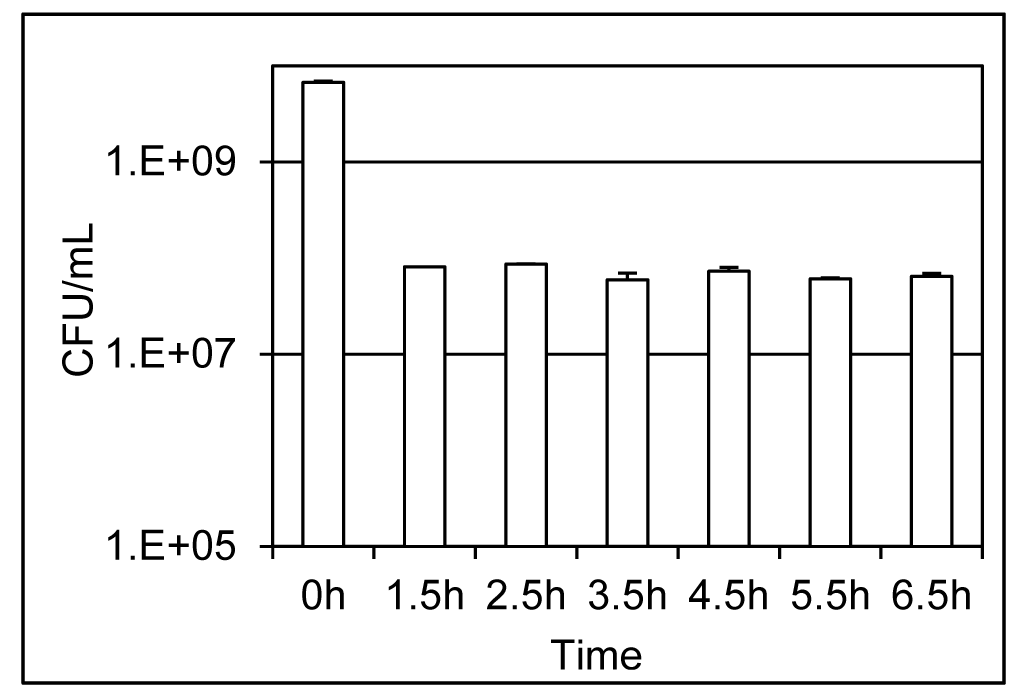

Supplement: Figure S2 — Effects of Cip treatment time on PAO1 killing. An 18-h overnight culture of PAO1 was treated with 200 µg/mL Cip for different lengths of time to determine the required treatment time for persister isolation. (TIF) [file pone.0045778.s002.tif]

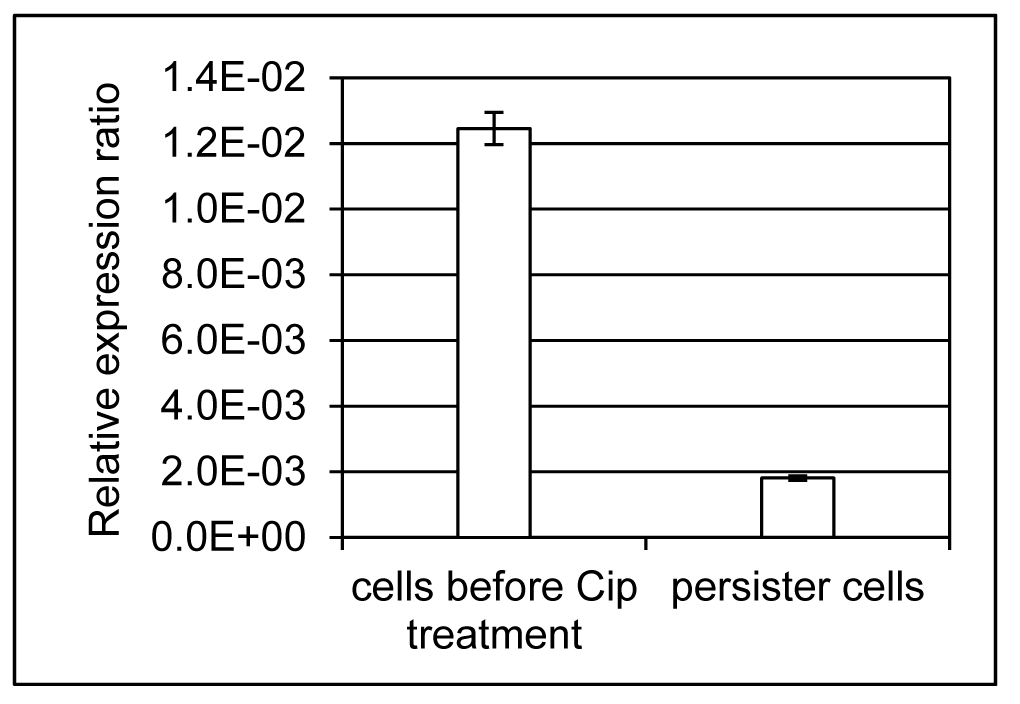

Supplement: Figure S3 — Transcription level of the housekeeping gene, proC , from total cells (before Cip treatment) and persister cells quantified with Q-PCR. The persister cells were isolated following the same procedure as described in the manuscript. The cells before and after Cip treatment were used to isolate total RNA and compare the transcription levels of proC. The persister cell sample was found to have 85.5% less proC compared to that of total cells before Cip treatment. (TIF) [file pone.0045778.s003.tif]
