# Supplementary material for: Reverting Antibiotic Tolerance of Pseudomonas aeruginosa PAO1 Persister Cells by (Z)-4-bromo-5-(bromomethylene)-3-methylfuran-2(5H)-one
Source: PLoS One. 2012 Sep 20;7(9):e45778. doi: 10.1371/journal.pone.0045778 (PMC3447867; doi:10.1371/journal.pone.0045778)
Supplement: Table S1 — List of BF8-inducded genes in PAO1 persister cells. A total of three biological replicates were tested. The genes induced by more than 2 fold in all three data sets are listed below. (DOCX) [file pone.0045778.s004.docx]

| **Induced gene** | **Expression ratio** | **Gene products/Functions** |
| --- | --- | --- |
| PA4167 | 510.6 | 2,5-diketo-D-gluconate reductase B |
| PA1334 | 227.6 | oxidoreductase |
| PA4173 | 36.7 | hypothetical protein |
| PA0182 | 97.1 | 3-ketoacyl-(acyl-carrier-protein) reductase |
| PA2932(*morB*) | 64.9 | morphinone reductase |
| PA0741 | 16.5 | hypothetical protein |
| PA1210 | 21 | hypothetical protein |
| PA3240 | 14.4 | hypothetical protein |
| PA3523 | 9.6 | Resistance-Nodulation-Cell Division efflux membrane fusion protein precursor |
| PA2535 | 9 | oxidoreductase |
| PA2575 | 9.1 | hypothetical protein |
| PA2931 | 11 | CifR |
| PA0565 | 12.8 | hypothetical protein |
| PA2580 | 8.3 | hypothetical protein |
| PA2610 | 7.2 | hypothetical protein |
| PA2839 | 11 | hypothetical protein |
| PA0422 | 4.2 | hypothetical protein |
| PA3223(*acpD*) | 4.8 | AzoR3, azoreductase 3 |
| PA1374 | 3.4 | hypothetical protein |
| PA3920 | 3.9 | metal transporting P-type ATPase |
| PA4878 | 4.2 | transcriptional regulator |
| PA1285 | 4.4 | transcriptional regulator |
| PA1470 | 4.1 | short chain dehydrogenase |
| PA3133 | 3.5 | transcriptional regulator |
| PA2196 | 4.8 | transcriptional regulator |
| PA2378 | 3.1 | aldehyde dehydrogenase |
| PA2691 | 3.7 | hypothetical protein |
| PA1127 | 3.4 | oxidoreductase |
