# Supplementary material for: Reverting Antibiotic Tolerance of Pseudomonas aeruginosa PAO1 Persister Cells by (Z)-4-bromo-5-(bromomethylene)-3-methylfuran-2(5H)-one
Source: PLoS One. 2012 Sep 20;7(9):e45778. doi: 10.1371/journal.pone.0045778 (PMC3447867; doi:10.1371/journal.pone.0045778)
Supplement: Table S2 — The primers used in RNA slot blotting and the blotting results. PA4943 was unchanged based on DNA microarray data. All the other 4 genes were induced by BF8 based on microarray results. DOCX [file pone.0045778.s005.docx]

| **Gene** | **Primers** | **Expression ratio based on RNA slot blot** |
| --- | --- | --- |
| PA4943 | GAAACGGTGGCATTCGTC | unchanged |
|  | GTTTCCAGCTGGGTCTCG |  |
| PA3523 | CCAGCAACTGTTCCTCATCG | 2 fold induction |
|  | CAGGTAGGTGCGCTCGTC |  |
| PA2931 | CGAGGCGATGGAAATCAG | 4 fold induction |
|  | GCATAGAAGGTCGCCAACTC |  |
| PA0182 | CGACATCCTGGTCAACAATG | 2 fold induction |
|  | GGTGATGTAGGCCGCTTC |  |
| PA4167 | GCAGATCTACGGCAACGAG | 3 fold induction |
|  | GCAAGTAAGGGCTGAGTTCG |  |
